# Current Trends, Future Prospects and Constraints of Whole Microalgae and Their Fractions as a Functional Feed Ingredient for Animals

**DOI:** 10.1111/jpn.70042

**Published:** 2026-01-14

**Authors:** Sietse Jan Koopmans, Simon Roques, Edoardo Zaccaria, Annemarie Mens, Soumya Kanti Kar

**Affiliations:** ^1^ Animal Nutrition Wageningen University and Research, Wageningen Livestock Research Wageningen the Netherlands; ^2^ Cuurent address: Universite Clermont Auvergne, INRAE, VetAgro Sub, UMR Herbivores Saint‐Genes‐Champanelle France

**Keywords:** bioactivity, fractions, functional feed ingredient, health, livestock, microalgae

## Abstract

Microalgae are a highly diverse group of unicellular organisms that grow in a wide range of aquatic environments and are widely used as dietary supplements for both human and animal applications. Microalgae are rich in lipids, proteins, carbohydrates and other valuable bioactive components such as pigments, antioxidants and vitamins. Those components have shown bioactivity not only by affecting cell, organ and tissue functionality but also have notable antimicrobial and immunomodulatory properties, positioning microalgae as a potential natural antibiotic substitute. Although production costs of microalgae are high, it has been shown that relatively low (< 1%) inclusion levels of microalgae in the diet of animals affect physiological functions and performance. Microalgae can be fed as whole biomass but also as a fraction (the lipid, protein, carbohydrate or rest fraction). Feedings a fraction of microalgae may be beneficial when only a specific bioactivity of a fraction is required in animals, thereby reducing the cost of feed. For instance, when microalgae are fractionated for human applications, the resulting byproducts or ‘side‐stream fractions’, present a cost‐effective feed alternative for livestock. In addition, feeding microalgae or their fractions during periods when young animals are more susceptible to health issues can not only enhance cost‐effectiveness but also potentially support their recovery. The aims of this review are (i) to present an overview of the mode of action of the lipid, protein and carbohydrate (rest) fractions of microalgae on whole body physiology, (ii) to summarize previous research on the bioactivity of dietary fractions of microalgae in livestock production and (iii) to propose novel strategies to use whole microalgae biomass or their fractions as functional feed to support resilience in young growing animals during vulnerable health episodes.

## 1 Introduction

Most feed for monogastrics such as chickens and pigs derives from terrestrial resources such as soybeans, corn, wheat and oilseed. Therefore, livestock production directly competes with crop production for land and resources (Karlsson and Röös 2019). Given global warming and climate change threats, animal nutritionists are currently emphasizing local resources over imported feed ingredients. This combined with the EU‐ban on feeding antibiotics (Millet and Maertens 2011) and pharmaceutical dose of ZnO (European Parliament 2001; European Parliament 2004) increases the importance to produce diets that maintain and/or improve animal performance and promote resistance to pathogens. In addition to an increase in animal performance and health, the quality of the animal products for human consumption is also important. Consumers expect animal products to have high nutritional value and quality in meat, eggs and milk (de Araújo et al. 2022), so production and quality are inextricably linked.

Feeding chickens, pigs and cattle to produce enough meat, eggs and milk to feed more than seven billion people is a great challenge. Maintaining such a rate of production while keeping our ecosystem and biodiversity in balance is becoming an even greater challenge (Herrero et al. 2015). With livestock production expected to double between 2017 and 2050 (Hoque et al. 2022), a significant expansion in the cultivation of land‐grown commodities will be required to meet nutritional needs in the livestock sector. Alternatives to land‐grown commodities such as soybean, corn, wheat and oilseeds are needed to maintain the balance between food, feed and biofuel industries. Soil degradation, water scarcity and drastic climate change are also major challenges for livestock agriculture. Accordingly, novel sustainable feedstuffs and improved resource efficiency will play a critical role in the sustainability of livestock systems on arable land. Microalgae could partly fulfil this need because of their non‐reliance on land, coupled with sustainable cultivation possibilities in a wide range of highly‐controlled aquatic settings (Olabi et al. 2023), which positions them as potential breakthrough.

Beyond their strict nutritional value, feed ingredients can also provide ‘non‐strict nutritional’ functional properties that influence factors such as feed intake (satiety), gastrointestinal passage rate, pro‐ and antimicrobial activity, antioxidative effects, immune signalling and metabolic responses (Biesalski et al. 2009; Lallès et al. 2009; Jansman 2016). The potential of microalgae as a feed ingredient for poultry, pigs and cattle has been increasingly recognized (Saadaoui et al. 2021; Bature et al. 2022; Van Nerom et al. 2024). Their role, however, depends strongly on the level of dietary inclusion. At lower doses (generally < 1%), microalgae are mainly considered as a feed additive due to their bioactive compounds (e.g., pigments, antioxidants, fatty acids, vitamins), which can modulate animal physiology, immune response and health (Roques et al. 2022). These bio‐functional effects enhance the economic attractiveness of microalgae in livestock feeds.

In contrast, at higher inclusion levels (2%–10%), microalgae function more as a nutritional ingredient, particularly as a protein source. This is especially relevant for protein‐rich species such as *Chlorella* or *Spirulina*, where the contribution to dietary protein supply becomes nutritionally significant, alongside lipids and carbohydrates. Their use at these higher inclusion levels may gain further importance if cultivation costs decrease. Nevertheless, both the strict nutritional and the non‐strict nutritional functional properties of microalgae are highly variable. This variability depends on (i) the specific species and its chemical composition (protein, lipids, polysaccharides, vitamins, pigments, antioxidants and minerals), (ii) production conditions (time of year, pH, temperature, cultivation method) and (iii) the specific nutritional requirements of the animal species, their life stage and health status (Madeira et al. 2017; Alagawany et al. 2021). Microalgae can be supplied as whole biomass or as specific fractions (lipids, proteins, carbohydrates or other components). Targeting a particular fraction for its bioactive properties may be advantageous for specific animal needs, potentially reducing overall feed costs.

It is essential to evaluate the chemical composition of microalgae not only in terms of their nutritional value but also for their functional properties. This review examines the primary macronutrient fractions of microalgae—namely proteins, lipids and carbohydrates—and their impacts on animal performance and health. It will explore the potential applications of microalgae based on the functional roles of these macronutrient fractions, tailored to different animal species, life stages and health conditions. In the absence of comprehensive studies on fractionated microalgae constituents, this review cites total biomass studies as proxies to illustrate potential biofunctional effects, although direct validation remains limited. Additionally, the review will address the cost‐effective use of microalgae byproducts as functional feed ingredients and discuss strategies for incorporating whole biomass or fractionated microalgae, particularly during critical health phases such as the neonatal, weaning and post‐calving periods.

## 2 Bioactive Microalgae Fractions

Microalgae and their derived fractions present valuable sources of bioactive compounds suitable for enhancing animal feed, both in livestock and in the young pet segment. The process of biorefinery facilitates the extraction of these fractions by breaking down the biomass into distinct macro‐ and micro‐nutrient components. While this review primarily focuses on biofunctions of various microalgae fractions and compounds in animals, comprehensive discussions of biorefinery techniques are available in the works of other authors (Vanthoor‐Koopmans et al. 2013; Ba et al. 2020; Kuo et al. 2021), which complement the perspectives presented here. Although this process can enhance the value of the final products, it also raises production costs. As a potential solution, utilizing by‐products from biofuel production or other human applications (Ibrahim et al. 2023) could provide a cost‐effective source of microalgae fractions for animal feed, while optimizing biorefinery techniques to yield nutrient‐rich fractions from the whole microalgae biomass. Recent studies also emphasize that despite higher investment costs compared to commercially available counterparts derived from traditional agricultural sources, microalgae systems offer long‐term economic and environmental benefits as they reduce dependence on arable land and freshwater (Ferreira et al. 2025). Redirecting research efforts from biofuel applications to biorefinery co‐ and by‐products is crucial to improve the economic viability and competitiveness of microalgae‐derived fractions (Bhattacharya and Goswami 2020). Biorefineries fractionate whole microalgae biomass, isolating various macro‐ and micro‐nutrient‐rich components. The macronutrient fractions encompass the lipid or oil fraction, the protein fraction and the residual fraction containing carbohydrates and ash. The micronutrients—pigments, vitamins and minerals—may be present in different concentrations in the macronutrient fractions (Pignolet et al. 2013; Bastiaens et al. 2017). Various techniques for extracting microalgae fractions include solvent‐based lipid extraction, aqueous solubilization for proteins and obtaining carbohydrate‐rich residues following lipid and protein extraction (Chew et al. 2017). However, to enhance the absorption and utilization of bioactive compounds from microalgae, purification is often necessary, as undigestible macromolecules in the cell walls (e.g., cellulose, hemicelluloses, sulfated polysaccharides, algaenan/sporopollenin‐like polymers and callose) can obstruct their effectiveness in humans or animals. To address this issue, milling and disrupting the whole microalga prior to fractionation have proven effective (Mendes‐Pinto et al. 2001; Neumann et al. 2018; Teuling et al. 2019).

In Table 1, the health benefits and biofunctionalities of chemical compounds present in the lipid, protein and residual fractions of microalgae are summarized. These functional ingredients are noted for their antimicrobial and immunomodulatory properties, suggesting their potential as natural alternatives to antibiotics (Gadde et al. 2017; DewiI et al. 2018; Saadaoui et al. 2021). Recent reviews have highlighted the significant health benefits of dietary microalgae, emphasizing their antioxidant and antiviral properties. Additionally, microalgae are recognized as promising candidates for animal feed due to their content of essential biomolecules, including amino acids, polyunsaturated fatty acids (PUFAs) and high‐value products such as carotenoids and vitamins (Holman and Malau‐Aduli 2013; Kotrbáček et al. 2015; Gadde et al. 2017; Saadaoui et al. 2021). The link from dietary fraction ingestion to the biological effects in livestock is often assumed and extrapolated from the in vitro and in vivo studies cited below (Table 1 and the following paragraph ‘Fractions of microalgae as functional feed ingredient’). With respect to in vitro research, it always remains uncertain whether orally ingested bioactive compounds of microalgae reach their targets in the body (certain cell types, microbiota and other biochemical compounds) and are able to exert their mode of action. With respect to previous in vivo research in non‐livestock species, it may be that certain biological effects of microalgae are species‐specific. In both cases, the cited in vitro and in vivo studies need to be validated for use in livestock species.

**Table 1:** Biofunctions of various microalgae fractions and compounds.

Fraction	Compounds	Effect/biofunctionality	Exhibits the function by modulating bio‐molecular pathways	References
Lipid	Xanthophylls including astaxanthin	Anti‐inflammatory	iNOS, COX‐2, TNF‐α, NFκB, interleukins	Choi (2019); Le Goff et al. (2019)
	PUFA, oxylipins	Anti‐inflammatory	PPARα/γ receptor	Chou et al. (2008); Ávila‐Román et al. 2018
	Phytosterols	Anti‐inflammatory	iNOS, COX‐2, TNF‐α, NFκB, interleukins	Ciliberti et al. (2019); Le Goff et al. (2019)
	Xanthophylls and phytosterols	Glucose‐lipid regulation	GLUT4, FAS, CY 36, PPARα	Le Goff et al. (2019)
Protein	Peptides	Anti‐inflammatory	NO, iNOS, NF‐κB, TNF‐α, PGE2, MDA, IL‐10, ROS, IL‐8, IL‐6, MCP‐1	Li et al. (2019)
	Peptides	Anti‐oxidant	Free radical scavenging	Sheih et al. (2009)
	Peptides	Glucose‐lipid regulation	Involved in the sensitivity of tissues to insulin regulation; DPP‐IV; regulation of the expression of genes related to lipid metabolism (SREBP‐1, ACC, PPARγ, AMPK, PPARα)	Hua et al. (2018)
	Peptides	Anti‐hypertensive	By acting on angiotensin I converting enzyme (ACE) enzyme	Yamaguchi et al. (1989); Suetsuna and Chen (2001); Samarakoon et al. (2013); Wu et al. (2014); Xie et al. (2018)
	Peptides	Modulate gut microbiota	Enriched the abundance of ‘beneficial bacteria’ with health benefits that include *Peptococcaceae, Prevotella, Alistipes, Porphyromonadaceae, Barnesiella* and *Parasutterella*	Hua et al. (2018)
	Peptides	Wound healing	Stimulates collagen deposition, skin appendages and evidence of basal laminae and suppresses fibroblast and inflammatory cells	C. L. Chen et al. (2011); K. H. Kang, Qian, et al. (2013); Mohseni et al. (2019); de Melo et al. (2019)
Carbohydrate or residual fraction	Polysaccharides	Anti‐inflammatory	IFN‐γ and IL‐2 in PBMC	Mirzaie, Tabarsa, et al. (2020)
	Polysaccharides	Glucose‐lipid regulation	ACC, HMG‐CoA, SREBP‐1c gene expression	Wan et al. (2020)
	Polysaccharides	Modulate gut microbiota	Enriched the abundance of *Coprococcus_1, Lactobacillus* and Turicibacter and the concentrations of acetate, propionate and butyrate	Wan et al. (2020)

## 3 Fractions of Microalgae as Functional Feed Ingredient

### 3.1 Lipid Fraction

The biorefinery of microalgae is mostly oriented towards biofuel production and usually involves the extraction of the lipid fraction, particularly the glycerides (Chew et al. 2017; Khoo et al. 2023). Eventhough the lipid fraction is mostly used as biofuel, there are potential benefits for animals as well. The long‐chain fatty acid profiles of some microalgae species are enriched with PUFAs such as eicosapentaenoic acid (EPA), alpha‐linolenic acid (ALA), arachidonic acid (AA), docosahexaenoic acid (DHA) and linoleic acid (LA). These omega‐3 and omega‐6 fatty acids are considered essential, thus cannot be synthesized by humans and animals, and must be obtained through the diet. Beyond their essential nature, DHA and EPA are recognized for their biological functions that include anti‐oxidant and anti‐inflammatory activities, bolster mental health and mitigate risks associated with several ailments, including cardiac diseases, arrhythmia, stroke, rheumatoid arthritis and hypertension (Udayan et al. 2017).

Regarding bioactivities, PUFAs are also ligands for free fatty acid receptors, which are involved in the regulation of insulin secretion, insulin sensitivity, palatability, pain and inflammation. Also, PUFAs such as linolenic acid and linoleic acid decrease inflammation and improve metabolic diseases (Kimura et al. 2020). Chou et al. (2008) have demonstrated a high binding affinity of PUFAs derived from *Chlorella sorokiniana* for the peroxisome proliferator‐activated receptors (PPARs). These receptors are highly involved in controlling fatty acid oxidation, lipogenesis, glucose homoeostasis, insulin sensitivity, inflammation and cell proliferation and apoptosis (Kersten et al. 2000; Tyagi et al. 2011).

The activation of PPARs can be modulated by various lipid compounds present in microalgae, including astaxanthin (Choi 2019; Le Goff et al. 2019), oxylipins, lipid mediators resulting from PUFA oxidation (Ávila‐Román et al. 2018) and phytosterols (Ciliberti et al. 2019; Le Goff et al. 2019). Ávila‐Román et al. (2018) demonstrated that oxylipins extracted from *Chlamydomonas debaryana* and *Nannochloropsis gaditana* exhibit anti‐inflammatory effects on a human acute monocytic leukaemia cell line. In the inflammatory response, TNF‐α receptors activate nuclear factor κB (NFκB), which translocates to the nucleus and initiates the expression of various inflammatory mediators such as interleukins, nitric oxide synthase and cyclooxygenase‐2. Oxylipins from microalgae act as ligands for PPAR‐γ, and during its nuclear translocation, PPAR‐γ interacts with NFκB, thereby inhibiting NFκB's nuclear migration and subsequently reducing inflammatory responses (Ávila‐Román et al. 2018). Similarly, Ciliberti et al. (2019) observed that sterols from the lipid extract of *C. sorokiniana* bind to PPARs, leading to decreased proliferation of sheep peripheral blood mononuclear cells, which are crucial for immune response and inflammation regulation. Furthermore, Le Goff et al. (2019) reviewed the effects of xanthophylls and phytosterols on PPAR‐mediated functions, noting that astaxanthin‐bound PPAR‐γ modulates the expression of glucose transporter 4 (GLUT4), facilitating glucose uptake into muscle and adipose tissues. Phytosterols also influence lipid metabolism by regulating hepatic hydroxymethylglutaryl‐CoA reductase, which synthesizes cholesterol precursors, and by stimulating low‐density lipoprotein (LDL)‐C receptors to enhance the clearance of plasma cholesterol.

Besides modulating immune functions, the lipid fraction of microalgae has been linked with other biofunctionalities. Dembitsky et al. (2000) have characterized fatty acid amides from *Rhizoclonium hieroglyphicum* which as been recognized as ligands for the cannabinoid receptors in mammals. The receptors are mainly situated in the brain affecting behaviour, including mood, memory, appetite and pain perception, but also in the gut, where they modulate motility, enteroendocrine functions and intestinal barrier functionality (Zou and Kumar 2018). Furthermore, Toraman et al. (2016) identified several nitrogen related metabolites in microalgae bio‐oils, such as indoles, amides, amines and imides, with roles in muscle contraction, behaviour modulation, gut motility and antioxidant activities (Tomberlin et al. 2017).

In conclusion, the lipid fraction of microalgae, rich in essential PUFAs and other bioactive compounds, holds significant potential beyond biofuel applications. Its diverse benefits, including anti‐inflammatory effects, metabolic improvements and modulation of immune responses, highlight its promise in enhancing both animal health and human nutrition.

### 3.2 Protein Fraction

For cost‐efficiency, defatted microalgae are often used rather than separately extracting the protein fraction. However, cell wall disruption techniques employed during lipid extraction can compromise protein integrity, making direct protein extraction a better option when an intact protein hydrolysate is needed. Although protein hydrolysate has already been produced from various sources such as feather meal (Pan et al. 2016), fish meal (Nørgaard et al. 2012) and insect meal (Cho et al. 2020) and used as such in livestock feed, microalgae's potential remains relatively untapped despite the existing research. For example, a study on undernourished mice demonstrated that dietary supplementation with *Chlorella vulgaris* enzymatic hydrolysate positively affected hematopoiesis and leucocyte count (Morris et al. 2007). The undernourished mice showed a decrease in immunocompetence due to decreased bone marrow cellularity. However, the *C. vulgaris* hydrolysate was found to recover bone marrow cellularity and increase the number of peritoneal exudates immuno‐potential cells, while a commercial diet had no effect.

Peptides derived from microalgae have a broad range of applications, including anti‐inflammatory, antioxidant, antidiabetic, antihypertensive and dyslipidemia‐reducing effects (Li et al. 2019). However, the bioavailability of these peptides after digestion remains uncharacterized as most information was obtained from in vitro or in vivo and involved parenteral administration. Peptides from *C. vulgaris* have been shown to reduce lipopolysaccharide (LPS)‐induced inflammation in an in vitro study using macrophage cells and in vivo through the topical application on thermally injured rats (Cherng et al. 2010). The peptides reduced pro‐inflammatory cytokines such as TNF‐a in injured rats. The anti‐oxidant effect of peptides derived from *C. vulgaris* was demonstrated in vitro by quenching free radicals and protecting the DNA (Sheih et al. 2009), while dietary phycocyanin derived from *Spirulina platensis* improved growth and antioxidant/anti‐inflammatory indices in growing rabbits under heat stress (Abdelnour et al. 2020). The anti‐hypertensive activity of *C. vulgaris* selected peptides was tested in rats, resulting in the reduction of diastolic and systolic blood pressure by the tri‐peptides Thr‐Thr‐Trp and Val‐His‐Trp, respectively, 2 h after oral gavage (Xie et al. 2018). It was suggested that the mode of action of these peptides was by binding to angiotensin converting enzymes (ACE) (Xie et al. 2018). Noteworthy, microalgae's anti‐hypertensive effects are widely accepted and were described as early as 1989 (Yamaguchi et al. 1989). The presence of ACE inhibitory peptides has been shown in frequently used microalgae species like *C. vulgaris* and *S. platensis* (Suetsuna and Chen 2001), in *Nannochloropsis oculata* (Samarakoon et al. 2013) and *Isochrysis* spp. (Wu et al. 2014; J. Chen et al. 2020). In addition, the dyslipidemia‐reducing‐activity of peptides derived from *S. platensis* has been shown in rats (Hua et al. 2018). Peptide containing protein hydrolysate reduced expression of genes related to lipogenesis, such as sterol regulatory element‐binding protein 1c (SREBP‐1c) and acetyl CoA carboxylase (ACC), which participate in the synthesis of free fatty acids. Peptides derived from microalgae also offer some unique applications for wound healing and regulation of fibroblast functionality, as demonstrated by several studies on *C. vulgaris* (C. L. Chen et al. 2011; K. H. Kang, Salim, et al. 2013; Mohseni et al. 2019; de Melo et al. 2019). Although the mode of action is not entirely deciphered, the most common hypothesis is that the peptides from the microalgae modulate the expression of *matrix metalloproteinase‐1*, which is responsible for collagen degradation.

In summary, peptides derived from microalgae exhibit notable biofunctional properties such as anti‐inflammatory, antioxidant, antihypertensive and dyslipidemia‐reducing effects. Despite promising in vitro and in vivo findings, further research is required to assess their bioavailability and underlying mechanisms. While defatted microalgae offer cost‐efficiency, direct protein extraction is recommended to maintain peptide functionality, especially for these biofunctional properties.

### 3.3 Carbohydrate or Residual Fraction

The residual fraction that remains after microalgae's lipid and protein extraction, is composed of carbohydrates and minerals. The bio‐activity of microalgae derived polysaccharides is greatly influenced by their structural features, such as molecular weight, monosaccharide compositions, glycosidic bonds and functional groups (sulfate and acetyl groups) (Yuan et al. 2020). In addition, the bio‐activity differs depending on the microalgae species and type of fractionation imposed on microalgae for deriving the polysaccharides. The carbohydrate fraction of microalgae mainly consists of the polysaccharides from the cell walls. The biological activities induced by the polysaccharides are anti‐microbial, immunomodulatory, anti‐oxidant, hypolipidemic, anti‐cancer and anti‐asthmatic (Yuan et al. 2020; Tounsi et al. 2022). Wan et al. (2020) extracted and purified a polysaccharide from *Chlorella pyrenoidosa* and showed its hypolipidemic activity using a rodent model. Additionally, the study showed that the bioactive compounds increased short‐chain fatty acids suggesting that this increase directly resulted from the modulation of the gut microbiota composition with increased abundance of bacteria like *Coprococcus*, *Lactobacillus* and *Turicibacter*, which are often associated with improved health (Wan et al. 2020). Polysaccharide extracts of *C. vulgaris* have shown immunomodulatory effects on peripheral blood mononuclear cells in chickens (Mirzaie, Tabarsa, et al. 2020). Both crude polysaccharides and extracts of polysaccharides increased the expression of systemic cytokines like interferon‐gamma and interleukin‐2 in chicken, demonstrating the immunomodulatory potentials of polysaccharides like ß‐glucans derived from microalgae (Mirzaie, Tabarsa, et al. 2020). As yet, little information is available on the bioactivity of extracted carbohydrates and polysaccharides from microalgae, and most knowledge has been gathered by extrapolation from the whole biomass of microalgae. Tounsi et al. (2022) have summarized the gathered knowledge on polysaccharides derived from microalgae and indicated the fibre‐source potential, the prebiotic action and the ability to promote beneficial intestinal bacteria as a functional food.

In summary, the residual fraction of microalgae contains specific carbohydrates, especially the bioactive polysaccharides, which makes it an attractive feed ingredient with potential biofunctions for livestock species, as reviewed by Tounsi et al. 2022.

## 4 Fractions of Microalgae Used in Livestock Feed

A summary of livestock studies on the lipid fraction, the defatted protein‐rich and defatted carbohydrate‐rich fractions of microalgae is presented in Table 2.

**Table 2:** Summary of research studies on microalgae fractions used in livestock animals.

Fraction	Microalgae species	Livestock species	Objective	Conclusions	References
Lipid fraction	*Spirulina platensis*	Poultry	Improve the egg's lipid profile	Increase DPA temporarily during the trial but the effect is not maintained	Michalak et al. (2020)
	Not defined	Cattle	Assess the feasibility of lipid encapsulation of microalgae oil to improve the n‐3 content of milk	Improvement of milk n‐3 content	Stamey et al. (2012)
	*Schizochytrium* sp.	Sheep	Assess whether supplementation of DHA‐rich algal oil enriches milk n‐3 and improves milk‐fat health indices	Enriched milk DHA and total n‐3 and lowered SFA/atherogenic index, but reduced DMI, milk yield and fat content	Manso et al. (2022)
	Not defined	Sheep	Determine how a marine algal oil fraction and forage source affect the fatty acid profile of ewe milk and milk yield	Marine algal oil (with soybean oil) increased milk DHA/CLA	Reynolds et al. (2006)
	*Schizochytrium* sp.	Goat	Compare microalgae oil with fish oil (and their combination) on rumen fatty acids/fermentation and on milk composition, fatty‐acid profile and performance in dairy goats	Microalgae oil increased milk/rumen PUFA without affecting performance	Beyzi and Dallı (2023)
Defatted protein fraction	*Staurosira* sp.	Poultry	Replace a portion of soybean and/or corn meal diet	7.5% of microalgae decreased body weight during the first 3 weeks but not at the end of the 6‐week trial	Austic et al. (2013)
	*Desmodesmus* sp.	Poultry	Assess the effect of the microalgae fraction on productivity and metabolism	Improved feed efficiency	Ekmay et al. (2014)
	*Chlorella vulgaris*	Poultry	Assess the effects of Chlorella by‐product (CBP) on immune responses, antioxidant status and intestinal morphology	Improved immune responses and increased villi height and crypt depth of the jejunum but reduced feed intake	Mirzaie, Sharifi, et al. (2020)
	Desmodesmus sp.	Pigs	Assess the effect of the microalgae fraction on productivity and metabolism	Decreased plasma uric acid and urea nitrogen, no changes in production	Ekmay et al. (2014)
	*Spirulina platensis*	Rabbits	Test dietary phycocyanin extract as an antioxidant/anti‐inflammatory additive to enhance growth, oxidative status and gut health in growing rabbits.	Improved growth and antioxidant indices, reduced inflammatory markers and caecal pathogens under heat stress	Abdelnour et al. (2020)
Defatted carbohydrate fraction	Not defined	Pigs	Evaluate the effects of a partially de‐oiled microalgae extract on growth performance and health status	Affects N metabolism, shift from urinary to faecal N excretion	Urriola et al. (2018)
	Chlorella	Poultry	Assess the benefits of microalgae fraction on gut health of chickens when used in early nutrition	No change of the overall diversity in gut microbiota	Zhang et al. (2020)
	*Nannochloropsis oceanica*	Pigs	Assess microalgae residual fraction as an iron source	Microalgae residual fraction restored both iron levels and growth performances in anaemic weaning piglets	Manor et al. (2017)

From the studies presented in Table 2, it can be concluded that biologic effects of fractionated microalgae are demonstrable and that specific conclusions on individual studies can be drawn. However, general conclusions are hard to formulate due to the limited number of studies and the large variation in experimental set‐up, such as strain of microalgae, type of fraction, animal species and primary outcome parameters.

### 4.1 Lipid Fraction

The organic fraction of microalgae, obtained after biorefinery stages, predominantly consists of polar lipids, such as phospholipids and neutral lipids, including triglycerides and fatty acids (Pignolet et al. 2013). This fraction comprises a mix of saturated, monounsaturated fatty acids (MUFA) and PUFA. The fatty acid content and ratios are influenced by the microalgae species and their growth conditions (Pignolet et al. 2013; Bastiaens et al. 2017). Moreover, some lipophilic pigments (mainly carotenoids) with high added value for the livestock sector can also be present in the organic fraction (Halim et al. 2012; Nobre et al. 2013). However, only a few livestock studies have assessed the effects of the lipid fractions on animal performance and physiology as well as animal welfare and health.

In poultry nutrition, the entire biomass of microalgae is frequently used as a matrix to deliver its lipid content, not only due to cost‐effectiveness but also because of the extensive nutritional benefits that the biomass provides beyond just lipids, as noted by Świątkiewicz et al. (2015). Research in this area predominantly centres on the impact of whole microalgae biomass on egg quality (A. Mens et al. 2022). However, only a couple of studies have focused specifically on the lipid fraction from microalgae in laying hens (Neijat et al. 2016; Michalak et al. 2020). Neijat et al. (2016) found that dietary omega‐3 fatty acids were effectively incorporated into egg yolks. Michalak et al. (2020) investigated the effects of a lipid extract from *S. platensis* on the fatty acid profile of eggs, noting that while there was a temporary increase in docosapentaenoic acid levels in eggs 60 days after supplementation, this effect diminished by 90 and 120 days. In swine production, the focus on microalgae lipid content, particularly the PUFA profile, is primarily driven by the goal of enhancing meat quality through the retention of omega‐3 fatty acids in muscle tissues (Sardi et al. 2006; Madeira et al. 2017; Vossen et al. 2017; Moran et al. 2018; de Tonnac and Mourot 2018; Kalbe et al. 2019). These studies have consistently utilized whole microalgae biomass as the source of lipids. To date, there is a lack of research specifically exploring the effects of isolated microalgae lipid fractions in pig studies. In cattle, the supplementation of whole microalgae for their lipid content aims to improve meat (Madeira et al. 2017) and milk quality. As a potential drawback, it has been shown that the inclusion of dietary PUFA via administration of whole microalgae leads to decreased feed intake and reduced milk fat (Allen 2000; Boeckaert et al. 2008; Marques et al. 2019). However, one study investigated the isolated lipid fraction from microalgae and the effects on omega‐3 fatty acids composition of milk. In this study, Stamey et al. (2012) used a rumen‐protected microalgae oil for 7 days and showed a constant increase in DHA content in milk fat for 6 consecutive days, but when the isolated oil fraction was compared to whole microalgae biomass, the yield transfer of DHA was superior for the whole microalgae biomass. This indicates that whole microalgae may be more effective than the isolated lipid fraction. In dairy ewes, supplementing DHA‐rich algal oil increased milk DHA and total n‐3 and lowered SFA/atherogenic index, although at ~2.3% algal oil, it reduced feed intake, milk fat and yield (Manso et al. 2022). A separate study using marine algal oil in combination with soybean oil increased milk DHA and CLA, with forage‐dependent magnitudes and milk yield remained largely unchanged (Reynolds et al. 2006). In dairy goats, microalgae oil raised milk/rumen PUFA (increased CLA and trans‐11 C18:1) without impairing performance (Beyzi and Dallı 2023). Although lipid fractions have been studied mainly in terms of their impact on the quality of meat, eggs and milk, their influence on general animal health is still less researched. Therefore, further research is needed to evaluate the effects of specific fractions in addition to the whole biomass.

### 4.2 Defatted Protein‐Rich Fraction

From a nutritional perspective, the protein fraction may hold the greatest potential for livestock feed, as it remains the predominant component following lipid extraction. The defatted protein‐rich aqueous fraction of microalgae is certainly the most used fraction in animal feed, firstly because of the need for sustainable protein in the livestock sector and secondly, because the lipid fraction (the oil) of microalgae is often used for biofuel production or human application (Halim et al. 2012; Bastiaens et al. 2017; Amorim et al. 2021). In livestock feed research, the defatted microalgae fraction is primarily utilized to investigate the impact of dietary protein on animal performance and health. However, it is important to note that this fraction also contains carbohydrates and micronutrients, including various forms of protein (Bleakley and Hayes 2017).

In poultry, defatted microalgae *Staurosira* sp. has been used to assess the replacement of soybean meal and corn in both broilers and laying hens (Austic et al. 2013). Furthermore, defatted *Desmodesmus* sp. of microalgae was used to assess growth performance and protein metabolism of broilers (Ekmay et al. 2014). The crude protein content of defatted fractions of microalgae greatly varied among the studies. For instance, Austic et al. (2013) used defatted microalgae that contained 19.1% crude protein, whereas the content of crude protein in the study of Ekmay et al. (2014) was up to 31.2%. The effects of these defatted microalgae on growth performance were largely comparable to conventional protein ingredients, except for a decrease in body weight during the first week of life (Austic et al. 2013). Further, Mirzaie, Sharifi, et al. (2020) used *C. vulgaris* defatted fraction to assess broilers' immune responses, antioxidant status and intestinal mucosal morphology. The defatted microalgae fraction at a 1% dietary inclusion level seems to have beneficial effects on both the immune system and gut health by improving cell‐mediated immune responses and enhancing the structural integrity of the small intestine.

In swine and poultry, Gatrell et al. (2014) have studied four types of full‐fat and defatted microalgal biomass from biofuel production research that contain 13.9%−38.2% crude protein and 1.5%−9.3% crude fat. Supplementing these microalgal biomasses at 7.5% in the diets of weanling pigs, broiler chicks and laying hens showed no adverse effects on growth performance, egg production and quality, plasma and tissue biochemical indicators and/or faecal chemical composition. In pigs, Ekmay et al. (2014) have assessed the effect of defatted *Staurosira* sp. and *Desmodesmus* sp. as protein substitutes in feed. The *Staurosira* sp. reduced the daily gain and gain‐to‐feed ratio, probably due to the defatted microalgae's high ash and relatively low protein level. However, the defatted *Desmodesmus* sp. at a dietary inclusion level of 10% did not affect daily gain or feed intake but decreased plasma uric acid and urea nitrogen concentrations by 23%−39%, suggesting increased metabolic protein efficiency (Kohn et al. 2005). The latter could be useful in reducing nitrogen emissions.

Currently, defatted microalgae fractions are predominantly employed as a proxy for investigating the effects of microalgae protein on livestock health and performance. The use of purified microalgae protein fractions in livestock feed is minimal in poultry, swine and nonexistent in cattle, likely due to unclear benefits and high production costs.

### 4.3 Defatted Carbohydrate‐Rich Fraction

The carbohydrate fraction that remains after microalgae's lipid extraction contains polysaccharides, including various β‐glucans, which are of great interest to the livestock sector (Pignolet et al. 2013). De Jesus Raposo et al. (2015) reviewed the diverse applications of microalgae‐derived polysaccharides, highlighting their anti‐viral, anti‐bacterial and anti‐inflammatory properties. Carbohydrate‐rich microalgae fractions have been used both in pigs and poultry. Urriola et al. (2018) formulated feed with up to 20% defatted microalgae extract, which was 76% carbohydrates and 5.72% protein. This high inclusion level tended to raise liver ammonia and lower liver arginine and ornithine, suggesting a downregulation of the urea cycle in nursery pigs. The reduction in urea metabolism is likely caused by a shift from urinary nitrogen (N) excretion towards faecal N excretion (due to increased incorporation of N in the faecal microbiota). Dietary carbohydrates from microalgae may enhance gut microbial fermentation, leading to the incorporation of free nitrogen, produced from pigs' protein metabolism, into microbial protein rather than its excretion in urine as urea and ammonia. Incorporating free N in faecal microbial protein is more environmentally friendly than urinary excretion of N as urea and ammonia. Research on poultry by Zhang et al. (2020) involved injecting polysaccharides from *Chlorella* sp. in ovo and assessing the birds' microbiomes. The study found no significant changes in microbial diversity or short‐chain fatty acid levels in the caecal digesta. The effectiveness of this in ovo injection technique for influencing the microbiome later in life remains uncertain. Importantly, *Chlorella* sp.'s polysaccharides resemble yeast cell wall ß‐glucans (Pignolet et al. 2013), which have a proven record in modulating livestock gut microbiota (Kogan and Kocher 2007).

In addition to the polysaccharides, the residual fraction of microalgae might also be used as a source of minerals in livestock. For instance, defatted *Nannochloropsis oceanica*, at a 0.5% inclusion, was as effective as inorganic iron in recuperating the growth performance of anaemic pigs (Manor et al. 2017). With a comparable approach and similar result, *C. vulgaris* was used as a selenium source, but the whole biomass was used rather than the residual fraction. The whole biomass of *C. vulgaris* was compared to selenium from sodium selenite. The effects of *C. vulgaris* on growth performance, selenium concentration in breast meat and excreta, activity of glutathione peroxidase in meat and oxidative stability of meat in broilers were equal to or better than the effects of supplemental sodium selenite (Dlouhá et al. 2008).

## 5 Fractions of Microalgae and Animal Performance

The effects of dietary whole microalgae on pig, poultry, cattle and small ruminant performance have been reviewed before (Madeira et al. 2017; Saadaoui et al. 2021; Kusmayadi et al. 2021; Orzuna‐Orzuna et al. 2023; Boukrouh et al. 2025). The general consensus is that whole microalgae, depending on the inclusion dose and digestibility, improve body weight gain of animals based on the nutritional and functional characteristics of the microalgae. In small ruminants, inclusion of microalgae in the diet, mostly whole *Schizochytrium* biomass, consistently enriches milk/meat with long‐chain n‐3 PUFA without major performance penalties at moderate inclusion; a goat meta‐analysis reports unchanged milk yield but higher lactose/protein/fat yields and a more favourable milk FA profile, and a lamb meta‐analysis shows improved growth and possible meat quality (Orzuna‐Orzuna et al. 2023; Boukrouh et al. 2025). Studies on fractionated microalgae are limited; the few small ruminant trials with DHA‐rich algal oil confirm strong milk DHA transfer but at higher doses can depress feed intake and milk‐fat (Manso et al. 2022; Reynolds et al. 2006; Beyzi and Dallı 2023). A study in goats by Kyriakaki et al. (2024) showed that adverse effects of high doses of the PUFA‐rich microalga *Schizochytrium* on performance may be alleviated by a diet with higher forage inclusion.

The whole biomass of *Chlorella* contains, in general, 20%−40% carbohydrates, 20%−60% protein and 10%−20% fat (Saadaoui et al. 2021). Austic et al. (2013) and Ekmay et al. (2014) showed that the effects of defatted microalgae on the growth performance of broilers were largely comparable to conventional protein ingredients when added to the diets. Gatrell et al. (2014) studied the effects of supplementing defatted microalgal biomass at 7.5% in the diets of weanling pigs, broiler chicks and laying hens. Compared to a control diet, similar effects on performance were reported. Apart from that, whole microalgae also enhance the nutritional quality of animal products like meat, eggs and milk (Vossen et al. 2017; A. Mens et al. 2022; Stamey et al. 2012). However, studies using fractionated microalgae on animal performance and product quality are scarce. Studies on fractionated microalgae generally emphasize animal health and the mechanisms by which microalgae affect physiological and immunological processes, rather than focusing on animal performance. As a result, animal group sizes are typically small, leading to low statistical power regarding growth and feed intake data. However, some research has documented effects of fractionated microalgae on animal performance. H. K. Kang et al. (2017) conducted a study in broilers which indicated that increasing the inclusion level of *Chlorella* by‐product by 25, 50 or 75 g/kg to a basal diet improved BW gain in a linear manner (*p* < 0.05). The *Chlorella* by‐product was low in carbohydrates (1.1%), protein (0.2%) and fat (0.9%), indicating that its impact on growth performance is likely due to functional rather than nutritional effects. Overall, these studies suggest that fractionated microalgae are well tolerated by growing animals and could serve as alternative nutritional or functional feed ingredients.

## 6 The Potential of Microalgae Fractions to Support Resilience in Animals

The increasing interest in microalgae biomass as an animal feed supplement is evident, but research on microalgae fractions remains limited. This is primarily due to the technical and cost‐related challenges of producing microalgae and their fractions. As production methods advance and the livestock industry continues to face challenges, there may eventually be a threshold where the cost of microalgae or its fractions becomes justified by the reduction in livestock losses or the benefit from livestock service. Incorporating whole microalgae as a supplement for young, growing animals during high‐stress periods, such as weaning, may provide an effective and cost‐efficient strategy to enhance livestock resilience and performance. As animals mature and their health becomes more manageable, the necessity for ongoing microalgae supplementation might decrease. Conversely, while maturation may reduce the need for continued supplementation, it is also associated with increased oxidative stress in older age. Therefore, supplementation with microalgae may remain beneficial at later stages of life due to their antioxidant properties. Moreover, using whole microalgae instead of isolated fractions could offer additional advantages, as they contain antioxidants that help protect bioactive components from degradation. For instance, the fatty fraction of microalgae, rich in PUFAs, is susceptible to oxidation when isolated. However, within the whole microalgae matrix, antioxidants like lutein and carotenoids can safeguard PUFAs from oxidation, thereby maintaining their beneficial effects on animal resilience (S. Lee et al. 2006). The ban on in‐feed antibiotics has increased the need for natural alternatives, such as dietary microalgae or their fractions, to replace antibiotics (Gadde et al. 2017; Saadaoui et al. 2021). To maximize the economic efficiency of microalgae as a functional feed, it is essential to identify specific time‐windows and vulnerable periods in animals' lives where microalgae inclusion can fortify resilience and long‐term health and development. For non‐mammalian animals, vulnerable periods typically occur in the weeks following birth, while for mammals, these periods are usually around weaning. In the case of broilers, the first 2 weeks after hatching are considered the most critical (Alagawany et al. 2021), while for laying hens, this vulnerable period for stress seems to be somewhere in the early rearing period before Week 10 (reviewed by A. J. W. Mens et al. 2020). For calves, vulnerable windows in which microalgae could be added to their (liquid) diet during the first 4 weeks after birth, while for other mammals such as pigs, dogs and cats and other ruminants, the 2‐week period after weaning is crucial (Heo et al. 2013; Wilson et al. 2017; Buddington and Sangild 2011). Furthermore, incorporating microalgae or their fractions into creep feed during the suckling phase may help ‘prepare’ mammals for the upcoming stressful and health‐compromising period that follows weaning (Xiong et al. 2019). A recent study (A. V. Lee et al. 2019) assessed the effect of PUFA's, especially DHA from the whole biomass of the microalgae *Aurantiochytrium limacinum*, on the immune‐ and global stress‐response of weaned piglets exposed to bacterial LPS. The microalgae inclusion in the diet reduced fever and cortisol levels in plasma and affected cytokine levels, indicating an immunomodulating effect of microalgae in young animals. Another effect of microalgae on young animals was reported by Manor et al. (2017), who showed that 0.5% of defatted *N. oceanica* restored the growth performance of anaemic weaned pigs. In young broilers (2−3 weeks of age), it was shown that the inclusion of 0.8% *C. vulgaris* biomass in the diet effectively influenced immune responses related to inflammatory status and promoted broiler growth (Roques et al. 2022).

In addition to these early life stages, microalgae could also serve during other critical periods in an animal's life when they are more susceptible to diseases and experience decreased performance. For instance, cows often face increased health challenges in the postpartum period due to the physiological demands of giving birth and the onset of lactation. Providing microalgae as a dietary supplement during this time may enhance the cow's immune system and overall health, improving productivity and reducing disease incidence. More generally, transition periods, such as changes in diet, housing or management practices, can create stress and negatively impact health and productivity in various livestock species. Microalgae supplementation during these times can help mitigate the negative effects of stress on animal performance, health and welfare. However, it is important to point out that immune‐related reactions to microalgae supplementation should be interpreted with caution. An observed stimulation of the immune system does not necessarily mean a positive or negative result. On the one hand, increased immune activity may reflect improved immune readiness, which contributes to disease resistance and resilience at critical times. On the other hand, prolonged or excessive immune activation can stress the metabolism and potentially divert resources from growth, reproduction or milk production. Therefore, immune responses should always be evaluated in the context of overall health, productivity and welfare indicators to determine whether the effect of microalgae is beneficial or a physiological burden.

The composition of (rest) fractions of microalgae is still a mix of macronutrients. For instance, the defatted protein fraction also contains some carbohydrate, and the defatted carbohydrate fraction also contains some protein. The lipid fraction may be the purest. All fractions show immune‐modulatory potential, and at the moment, it is not possible to indicate which fraction may be the most effective in supporting animal resilience at various challenges (inflammatory, oxidative stress, pathogen challenge). Therefore, studies in various livestock species are needed to determine the beneficial effects of whole microalgae and their (rest) fractions on the resilience of animals during vulnerable periods of their lives. To fully validate the applicability of microalgae as a supplement for young animals, it is essential to conduct comprehensive testing across various microalgae strains, growth conditions and substrates. This includes evaluating the effects of different microalgae fractions on a range of young animal species. Research should focus on identifying which strains offer the most significant benefits in terms of growth performance, resilience and overall health. Additionally, examining how varying growth conditions and substrates influence the quality and efficacy of the microalgae is crucial. This approach will help determine optimal conditions for microalgae cultivation and application, ensuring that the benefits observed in one species or setting can be consistently replicated across different species and environments. Such a thorough investigation will provide a robust foundation for implementing microalgae‐based supplements in diverse livestock systems.

## 7 Advantages and Disadvantages of In‐Feed Fractionated Microalgae

Advantages:

1. Optimal use of waste products (side‐streams after biorefinery of microalgae) thereby contributes to fulfilling governmental policies to improve resource efficiency and a circular economy.
2. Turn a low‐value waste product into a high‐value dietary supplement product.
3. Depending on the composition of the fraction, the desired bioactivity can be matched with the specific needs and phenotype of an animal.
4. Cell disruption techniques are necessary for fractionation, thereby releasing the bioactive compounds for optimal absorption by the gastrointestinal tract of the animal. Thus, this creates innovative opportunities for food/feed technologists or engineers.
5. Cultivation of microalgae is costly; therefore, low in‐feed dosages are mandatory for economic viability. This is possible because bioactivity occurs at low dietary inclusion levels (< 1%−2%). Fractionated microalgae as a side‐stream product of biorefinery further increases economic feasibility (achievability) for the use in feed.

Disadvantages:

1. The use of fractionated microalgae in feed is still in its infancy; large‐scale production is limited, and availability is low.
2. Feeding trials with fractionated microalgae are scarce; therefore, reliable information on the bioactivity of certain fractions of microalgae is not yet available.
3. Commercially, currently it lacks ‘value‐based pricing’ research that assesses how much customers are willing to pay based on the product's perceived value.
4. Standardization of the biorefinery process to fractionate microalgae on a large scale is lacking. Therefore, the composition and bioactivity of the fractions are variable and partly unpredictable.

## 8 Conclusions

While the utilization of whole microalgae biomass in livestock feed has been extensively explored, studies on microalgae fractions remain limited. Fractionation of the microalgae is costly, difficult to produce as bulk and therefore less used in feed. The biorefinery concept applied to microalgae supposes valorizing by‐products originating from biofuel production or human applications (food, pharma, cosmetics) for animal feed, which aids sustainability. However, this concept is still in its infancy and producing each fraction with satisfactory yield and quality seems, as yet, out of reach. As soon as by‐products from microalgae cultivation, like the defatted and deproteinized fractions, become available for animal feed in large quantities, these fractions of microalgae may become a cost‐effective alternative to the whole microalgae biomass. Until then, feeding whole microalgae biomass seems the best option to offer solutions for the livestock sector's challenges. Microalgae act as a functional feed on the animal's physiology, expressing anti‐inflammatory, anti‐oxidant, anti‐bacterial and anti‐viral bioactivity. Feeding whole microalgae biomass or fractions for restricted periods when (young) animals are vulnerable to stress and disease may be supportive to the animals' health and/or an alternative to in‐feed antibiotics and thus reduce the use of therapeutic antibiotics by creating resilient livestock. Microalgae or their fractions could be used in livestock species and pets as natural supplements for animals often facing pathogenic challenges. Relevant doses for microalgae or fractions thereof as a functional feed to increase health and resilience of young growing animals are likely to be below an inclusion level of 1%, thereby restricting the costs of supplementing animal feed with microalgae. Further dose‐response research is needed to characterize the fractions fully and to understand their in‐feed bio‐functionality and mode of action in vulnerable animals. It is also necessary to further test various microalgae strains, growing conditions, substrates and fractions thereof in various young animal species to validate applicability.

## Author Contributions

All authors contributed to the content and writing process of the review.

## Ethics Statement

The authors have nothing to report.

## Consent

The authors have nothing to report.

## Conflicts of Interest

The authors declare no conflicts of interest.

## Data Availability

Data sharing is not applicable to this article as no new data were created or analysed in this study.
